# Supplementary material for: No Changes in Body Composition and Adherence to the Mediterranean Diet after a 12-Week Aerobic Training Intervention in Women with Systemic Lupus Erythematosus: The EJERCITA-LES Study
Source: Nutrients. 2023 Oct 18;15(20):4424. doi: 10.3390/nu15204424 (PMC10609990; doi:10.3390/nu15204424)
Supplement: Supplementary file 1 [file nutrients-15-04424-s001.zip › nutrients-2649047-SI.pdf]

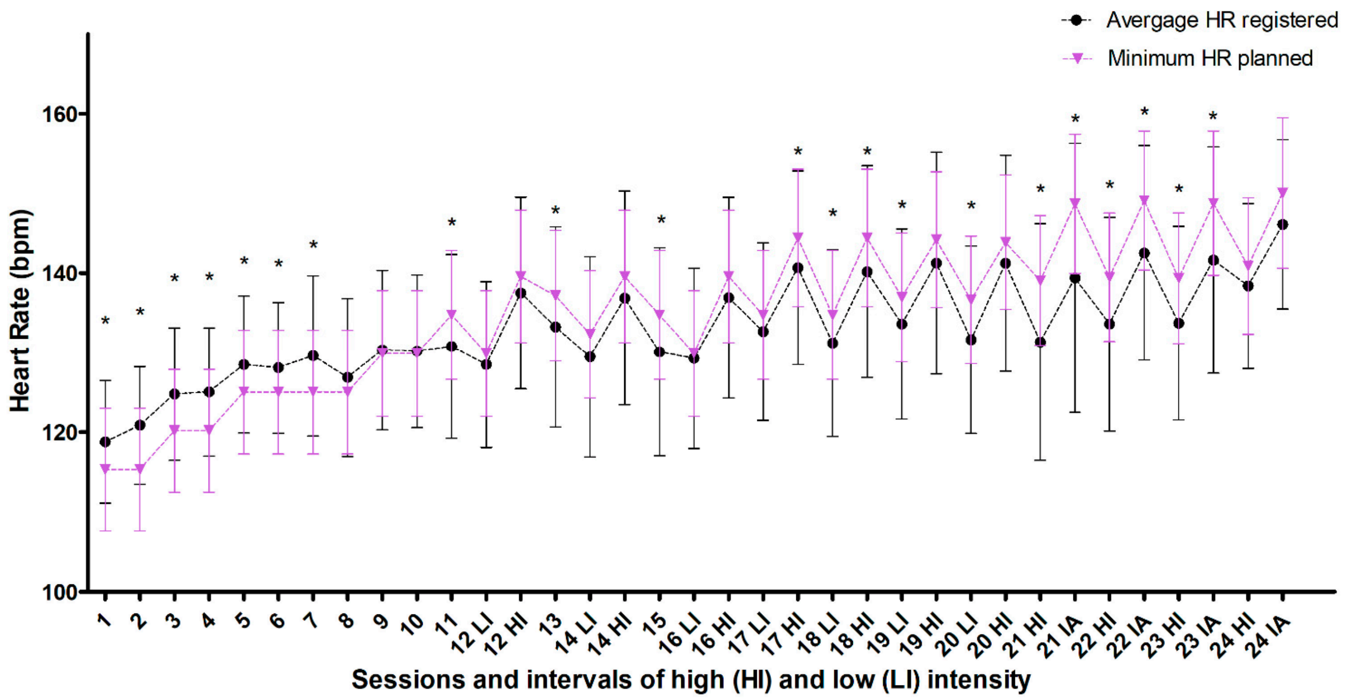

Supplementary Figure S1. Differences between average heart rate (HR) registered and minimum heart rate planned for sessions and intervals of the training program. Bpm: beats per minute, * $p < 0.05$
